# Genome sequence of *Trueperella pyogenes* isolates from liver abscesses in feedlot cattle

**DOI:** 10.1128/mra.00554-24

**Published:** 2024-11-11

**Authors:** Robert J. Gruninger, Rahat Zaheer, Matthew Stuart-Edwards, Athanasios Zovoilis, Tim A. McAllister

**Affiliations:** 1 Lethbridge Research and Development Centre, Agriculture and Agri-Food Canada, Lethbridge, Alberta, Canada; 2 Department of Biochemistry and Medical Genetics, University of Manitoba, Winnipeg, Manitoba, Canada; 3 Department of Chemistry and Biochemistry, University of Lethbridge, Lethbridge, Alberta, Canada; Department of Biological Sciences, Wellesley College, Wellesley, Massachusetts, USA

**Keywords:** liver abscess, cattle, feedlot, genome, *Trueperella pyogenes*

## Abstract

High-grain diets promote polymicrobial liver infections in cattle, commonly involving the bacterium *Trueperella pyogenes*. We have isolated *T. pyogenes* from the purulent material of abscesses and sequenced their genomes. These data enhance our understanding of the mechanisms underlying liver abscess development in cattle.

## ANNOUNCEMENT

Liver abscesses (LA) are polymicrobial infections, which result in significant economic loses and commonly involving *Fusobacterium necrophorum*, *Bacteroides* spp., *Trueperella pyogenes*, and several other gut bacteria (1–3). *T. pyogenes* is an opportunistic pathogen causing various infections in livestock including mastitis, uterine diseases, skin infections, and LA (4, 5). We have isolated *T. pyogenes* from two severely abscessed livers collected after slaughter at a commercial abattoir. Livers were obtained from crossbred beef yearling steers (*Bos taurus*) raised in a commercial feedlot in Southern Alberta, Canada (6). Animal experiments were approved by the Animal Care Committee of the Lethbridge Research and Development Centre (AUP #:1642).

Following surface sterilization, LAs were dissected with a sterile scalpel, and purulent material was inoculated into CBS (Columbia broth + 5% sheep’s blood; BD Difco). A dilution series was plated onto CBS agar and incubated anaerobically (85% N_2_, 10% CO_2_, 5% H_2_) for 48 h at 37°C. Isolates were randomly selected and preliminarily identified by a search of a partial 16s rRNA gene sequence (Sanger sequencing primers 27F 5′-AGAGTTTGATCCTGGCTCAG-3′ and 806R 5′- GGACTACHVGGGTWTCTAAT-3′) with blastn (7, 8). The 16s sequence from *T. pyogenes* NCTC 5224 (genebank UHHW01000002.1) had an identity of >99% to the 16s rRNA sequences from the two LA abscess isolates (9).

Following a minimum of three isolation rounds to ensure isolate purity, genomic DNA was purified with Genomic-tip 20/G according to the manufacturer’s instructions (QIAGEN, MD, USA). Short-read sequencing of a NexteraXT DNA library was performed on the Illumina MiSeq platform with MiSeq reagent V3, 2 × 300-bp PE kit according to the manufacturer’s protocol. Quality control of Illumina reads was conducted with trimmomatic v0.39 using default parameters (10). Nanopore sequencing libraries were prepared using ligation sequencing kit V9 without DNA fragmentation as directed (LSK-109, Oxford Nanopore, Oxford, UK). Long-read sequencing was performed with a PromethION using flowcell v9.4.1 as directed by the manufacturer (Oxford Nanopore, Oxford, UK). MinKNOW Core 23.04.6 was used for flowcell signal processing and base calling. Adapter trimming and read QC were performed by Guppy 6.5.7, and reads passing the default quality cutoff were assembled *de novo* with Flye v2.9.1 (11). Hybrid genome assemblies were generated with Unicycler v0.5.0 using default settings (12). Genome quality was assessed using Quast v.5.2.0 (13) and was annotated with the Protein Genome Annotation Pipeline v6.7 (14). Genome assembly and annotation results are shown in Table 1. Genome size, GC content, and gene number are consistent with the type strain, *T. pyogenes* NCTC 5224 (9).

**TABLE 1:** Genome sequencing, assembly, and annotation results

	*Trueperella pyogenes* 188	*Trueperella pyogenes* 212
Illumina reads/coverage	3,566,782/286x	3,520,772/266x
PromethION (bp) / coverage	64,246,871/28x	76,784,181/33x
Genome size (bp)	2,317,733	2,323,226
Contigs	4	1
N50	2,309,975	2,323,226
Completeness	100%	100%
GC content (%)	59.5	59.5
Predicted genes	2,126	2,115
Protein coding	2,054	2,043
tRNA:rRNA	47:6	47:9
Genome:	JBEXCK000000000	CP158015
Illumina reads:	SRX24625228	SRX24625229
Nanopore reads	SRR30575160	SRR30575159

## Data Availability

Genome Sequences are deposited in GenBank: PRJNA1114104. Short and long reads are available in SRA: SRX24625228, SRX24625229, and SRR30575160 and SRR30575159, respectively.
